# Time trends in social differences in nutrition habits of a Lithuanian population: 1994-2010

**DOI:** 10.1186/1471-2458-12-218

**Published:** 2012-03-21

**Authors:** Vilma Kriaucioniene, Jurate Klumbiene, Janina Petkeviciene, Edita Sakyte

**Affiliations:** 1 Lithuanian University of Health Sciences, Medical Academy, Faculty of Public Health, Health Research Institute, Kaunas, Lithuania

## Abstract

**Background:**

During the post-communist transition period, political, economic, and social changes affected the lifestyles of the Lithuanian population, including their nutritional habits. However, people of lower socio-economic position were more vulnerable to these changes. The aim of the present study was to evaluate the trends in selected food habits of the Lithuanian adult population by their level of education and place of residence from 1994 to 2010.

**Methods:**

The data were obtained from nine biannual cross-sectional postal surveys of Lithuanian health behaviours, beginning in 1994. Each survey used a randomly selected nationally representative sample of 3000 inhabitants aged 20-64 drawn from the population register. In total, 7358 men and 9796 women participated in these surveys. Questions about food consumption were included within all health behaviour questionnaires.

**Results:**

During the transition period, use of vegetable oil in cooking and the frequency of consumption of fresh vegetables increased, use of butter on bread decreased, and the proportion of women drinking high-fat milk declined. Lithuanians with higher education reported more frequent use of vegetable oil in cooking as well as daily consumption of fresh vegetables than those with a lower level of education. Consumption of high-fat milk was inversely associated with educational background. In addition, the proportion of persons spreading butter on bread increased with higher education level. The greatest urban-rural difference was observed in high-fat milk consumption. The increase in the use of vegetable oil in cooking, and the reduction of spreading butter on bread was more evident among less educated and rural inhabitants. Meanwhile, a greater proportion of the rural population, compared to urban, reduced their use of butter on bread. Daily consumption of fresh vegetables increased most among highly educated Lithuanians.

**Conclusions:**

The data from our study indicate beneficial dietary changes among the Lithuanian adult population. In general, those with a higher level of education had healthier food habits than those with low education. The educational gradient in analyzed food habits, except the use of vegetable oil, enlarged. A higher proportion of the rural population, compared to urban, reduced their usage of butter on bread. However, consumption of high-fat milk was greatest in the rural population. Our data highlight the need for future food and nutrition policies, as well as health promotion programmes, targeting the whole population, particularly those with lower education and living in rural areas.

## Background

Socio-economic differences in health have been reported in many countries [1-3]. It is well known that health inequalities depend on economic, cultural, psychosocial, environmental and lifestyle factors. Diet is one such factor, playing an important role in the development of chronic non-communicable diseases (NCD), such as cardiovascular diseases (CVD), some cancers, and obesity; therefore, social differences in nutrition habits may partly explain social inequalities in morbidity and mortality from NCD [4-6]. Many studies have shown that people of higher socioeconomic status tend to have healthier diets than lower-status groups [7-9]. Social and economic changes in a country may impact nutrition habits, socio-demographic differences in diet, and other health behaviours of populations [10,11].

In Lithuania, the transition period from a centralized-communist to a market-oriented economy was characterized by a widening gap between social strata [12]. Mortality rates decreased among the highly educated but increased among those with lower levels of education [13]. Life expectancy of rural population was shorter compared to urban population [14]. During the post-communist transition period, food prices increased dramatically [14]. People with lower socio-economic position were more vulnerable to inflation and subsequent declines in real income. Meanwhile, the availability and variety of foods increased considerably in Lithuania. Different kinds of vegetable oil and margarine, as well as fresh fruits and vegetables, became easily accessible. Furthermore, health promotion programmes and new food legislation were introduced. However, relatively little is known about how these changes influenced social inequalities in Lithuanian nutritional habits.

Since 1994, health behaviour monitoring has been carried out among the Lithuanian adult population. The monitoring data provide an opportunity to study the changes of socio-economic differences in health behaviours during the post-communist transition period. This study focused on dietary behaviours. Specifically, the use of vegetable oil in cooking and daily consumption of fresh vegetables were chosen as examples of healthy dietary habits, while the use of butter on bread and drinking high-fat milk were considered as unhealthy food habits.

The aim of the study was to evaluate trends in selected food habits by the level of education and place of residence from 1994 to 2010.

## Methods

### Study design

The data from the Health Behaviour Monitoring among the Lithuanian adult population were analyzed [11,15]. Since 1994, nine cross-sectional surveys have been carried out biannually. For every survey, a nationally representative random sample, aged 20-64, was drawn from the National Population Register. The sampling unit was the individual in all surveys; no measures were taken to substitute non-respondents. The sample consisted of 3000 individuals in 1994-2008 surveys, and 4000 in the 2010 survey. The questionnaires were sent by mail in April, with one reminder mailed to those who did not respond. Response rates were satisfactory in all surveys, ranging from 54 to 74% (64% in 1994, 69% in 1996, 64% in 1998, 74% in 2000, 64% in 2002, 62% in 2004, 59% in 2006, 61% in 2008, and 54 in 2010). In total, 7358 men and 9796 women participated in the surveys.

The Lithuanian Bioethics Committee approved all surveys.

### Socio-demographic characteristics

Education and place of residence were chosen as socio-demographic determinants. Level of education was determined by the following question: 'What is your education?' Possible answer choices were: 1) primary, 2) incomplete secondary, 3) secondary, 4) vocational school, 5) college, 6) university. For analyses, respondents were categorized into four educational groups: 1) primary or incomplete secondary education, 2) secondary school, 3) college or vocational school, and 4) university.

According to the administrative classification of places of residence, the respondents were grouped as living in cities, towns or villages. The addresses of respondents, obtained from the National Population Register, were used for determination of living place. Cities included the capital city and four largest cities, towns - the centers of municipalities and towns with population at least 2000 inhabitants, villages - small towns and countryside.

The characteristics of the study population are presented in Table 1.

**Table 1:** Distribution of study population (%) by socio-demographic variables (1994 - 2010)

Socio-demographic variables	Men (n = 7358)	Women (n = 9796)
Age		
20-34	32.5	31.0
35-49	37.4	36.7
50-64	30.2	32.3
Education		
Primary school or incomplete secondary	18.2	12.8
Secondary school	28.9	25.1
College, vocational school	34.7	36.8
University	18.3	25.6
Place of residence		
City	42.4	46.0
Town	27.3	28.7
Village	30.3	25.3

### Assessment of food habits

The questionnaire included several food-related questions. The type of fat used in cooking was identified by the following question: 'What kind of fat do you mostly use for food preparation at home?' This variable was dichotomized as vegetable oil users and others. The respondents were also asked what kind of fat they mostly spread on bread. The variable was divided into two categories: butter users and others. The group of 'butter users' included persons using butter or mixture of butter and vegetable oil. The type of consumed milk was assessed with the following question: 'What kind of milk do you usually drink?' Milk drinkers were divided into those who consumed high-fat milk (3.5% fat content and more) and others. The question addressing the weekly consumption frequency of fresh vegetables was included in the questionnaire beginning in 1996. This variable was dichotomized as daily users (6-7 days per week) and others.

### Statistical methods

Data were analysed using the statistical package SPSS (version 15). Analysis was performed separately for men and women. The data were weighted to match the age distribution of the Lithuanian population aged 20-64 in 2010. Normal approximation was used in calculation of 95% confidence intervals for standardized proportions.

The distribution of the population by education varied between surveys. In order to compare the nutrition habits by education, a ranking measure for educational level was constructed and used in the calculation of a Relative Inequality Index (RII) and its 95% confidence intervals [16]. The ranked variable was expressed as the cumulative proportion of each education level within the educational hierarchy, with 0 (low) and 1 (high) as the extreme values. The RII was estimated using logistic regression, controlled for age and place of residence, separately for men and women. The RII can be interpreted as the odds of having a certain food habit at the top end of the educational hierarchy compared to the lowest end of the educational hierarchy.

The effect of the place of residence on nutrition habits was evaluated using logistic regression analysis. All models were applied separately for men and women. The odds of having a certain food habit were calculated with adjustment for age and education. The reference group was respondents living in villages.

## Results

**Figure 1:**
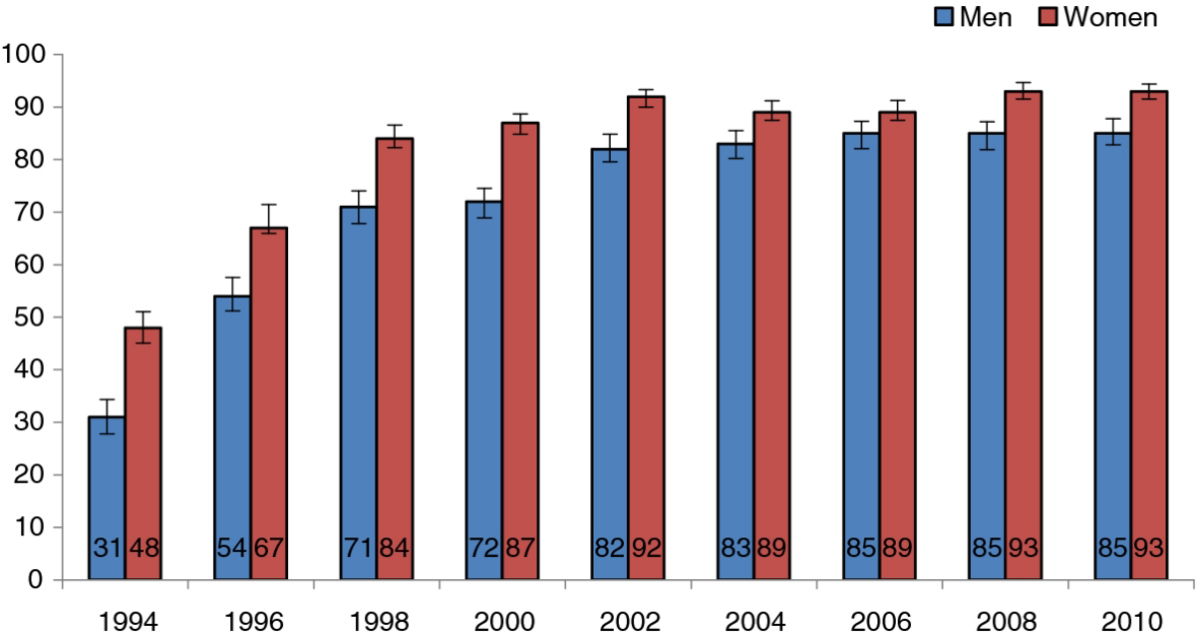
**Proportion of persons using vegetable oil in cooking in 1994-2010**.

Since 1994, the proportion of Lithuanian men and women using vegetable oil in cooking has risen. The largest increase was observed from 1994 to 1998. During this period, the proportion of persons using mostly vegetable oil in cooking increased by 2.3 times in men and by 1.8 times in women (Figure 1). In 2010, the majority of Lithuanian population, 85% (95% CI: 83-88) of men and 93% (95% CI: 92-94) of women, used vegetable oil in cooking.

**Figure 2:**
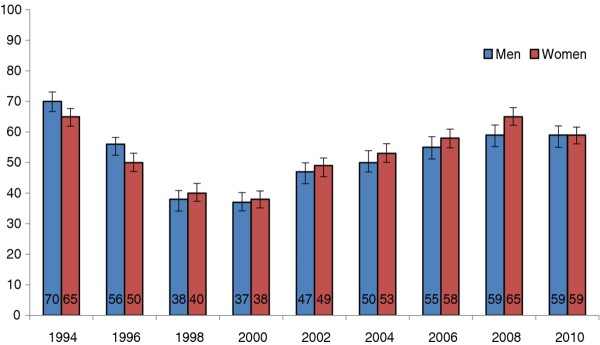
**Proportion of persons using butter on bread in 1994-2010**.

Between 1994 and 2000, the frequency of spreading butter on bread decreased from 70% (95% CI: 67-73) to 37% (95% CI: 34-40) in men and from 65% (95% CI: 62-68) to 38% (95% CI: 35-41) in women (Figure 2). Since 2000 though, the use of butter on bread increased. In 2010, 59% (95% CI: 56.5-60.9) of men and women reported spreading butter on bread.

**Figure 3:**
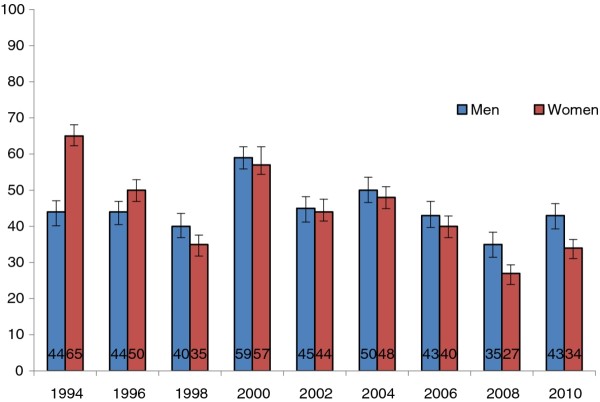
**Proportion of persons drinking high-fat milk in 1994-2010**.

The frequency of consumption of high-fat milk was high. However between 2000 and 2008, decreasing trends were observed. During this period, the proportion of people drinking high-fat milk declined from 59% (95% CI: 56-62) to 35% (95% CI: 32-38) in men and from 57% (95% CI: 42-60) to 27% (95% CI: 25-30) in women (Figure 3). The most recent survey, conducted in 2010, demonstrated that the proportion of men and women consuming high-fat milk had increased since the 2008 survey.

**Figure 4:**
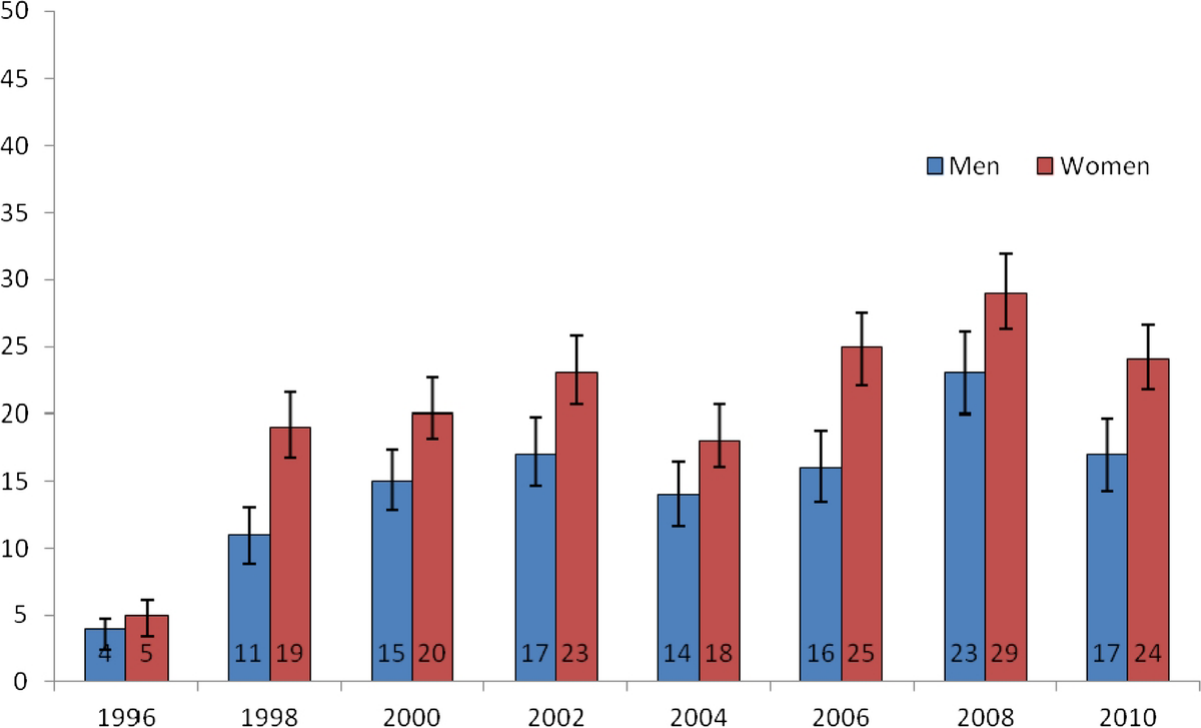
**Proportion of persons eating fresh vegetables daily in 1996-2010**.

Analysis of the trends in consumption of fresh vegetables between 1996 and 2008 showed that the proportion of daily consumers increased from 4% (95% CI: 3-5) to 23% (95% CI: 20-26) in men and from 5% (95% CI: 4-7) to 29% (95% CI: 26-32) in women (Figure 4). Despite this, a recent declining trend in the consumption of fresh vegetables appeared in the last survey. Since 1998, women reported more frequent consumption of fresh vegetables than men did.

Analyses of pooled data from all surveys showed nutrition habits were associated with education. People with higher education reported cooking with vegetable oil and daily consumption of fresh vegetables more often than the less educated (Table 2). However, consumption of high fat-milk was inversely associated with educational level. The proportion of persons spreading butter on bread increased with level of education.

**Table 2:** Age-adjusted prevalence (%) of given nutrition habits by educational level (95% confidence intervals) (all study years combined)

Nutrition habits	Education			
	Primary school or incomplete secondary	Secondary school	College, vocational school	University
**Men**	**n = 1336**	**n = 2124**	**n = 2549**	**n = 1342**
Using vegetable oil in cooking	59.1 (56.4-61.7)	73.2 (71.3-75.1)	72.7 (70.9-74.4)	78.4 (76.2-80.6)
Using butter on bread	47.2 (44.5-49.9)	49.7 (47.6-51.8)	51.2 (49.3-53.1)	59.4 (56.8-62.0)
Drinking high-fat milk	55.6 (52.9-58.3)	46.3 (44.1-48.4)	45.9 (44.0-47.9)	31.5 (29.0-34.0)
Eating fresh vegetables daily	9.0 (7.3-10.7)	14.6 (13.0-16.2)	14.7 (13.2-16.2)	18.3 (16.1-20.4)
**Women**	**n = 1251**	**n = 2456**	**n = 3607**	**n = 2476**
Using vegetable oil in cooking	63.9 (61.2-66.6)	83.8 (82.3-85.3)	83.5 (82.3-84.8)	89.9 (88.7-91.0)
Using butter on bread	48.1 (45.3-50.9)	45.8 (43.9-47.8)	53.8 (52.2-55.5)	60.4 (58.5-62.3)
Drinking high-fat milk	60.5 (57.7-63.2)	45.4 (43.4-47.4)	41.3 (39.6-42.9)	28.4 (26.6-30.1)
Eating fresh vegetable daily	13.5 (11.4-15.6)	17.1 (15.5-18.7)	20.2 (18.8-21.6)	26.1 (24.3-27.9)

Analyses of time trends in nutrition habits showed that use of vegetable oil increased in all educational groups, and that this increase was the largest among people with less education. Among men, educational differences in the use of vegetable oil vanished in 2000 (Table 3). In the majority of surveys, highly educated women used vegetable oil in cooking more often than the less educated. In 1994, the use of butter on bread was more common among less educated respondents (Tables 3, 4). In later years, the frequency of reported use of butter on bread decreased among less educated men and women. In contrast, the proportion of highly educated persons using butter remained stable among men and increased among women. Educational differences were significant in all surveys. During the study period, less educated people reported consumption of high-fat milk more often than the highly educated (Tables 3, 4). During the study, the increase in daily consumption of fresh vegetables was more evident among the highly educated compared to the less educated (Tables 3, 4). The greatest educational gradient was observed in 2000-2002 and 2010.

**Table 3:** Educational inequalities in nutrition habits among men (prevalence and RII*)

Years	Using vegetable oil for cooking			Using butter on bread			Drinking high-fat milk			Eating fresh vegetables daily		
	Education		RII	Education		RII	Education		RII	Education		RII
	Low	High	95% CI	Low	High	95% CI	Low	High	95% CI	Low	High	95% CI
1994	19.0	41.4	2.21 (1.20-4.07)	74.1	63.1	0.71 (0.39-1.29)	46.6	27.9	0.75 (0.41-1.39)	x	x	x
1996	42.0	66.2	1.20 (0.70-2.08)	55.0	65.5	1.60 (0.94-2.73)	48.6	31.7	0.91 (0.51-1.64)	2.7	2.8	1.40 (0.34-5.73)
1998	60.5	75.6	1.90 (1.04-3.39)	25.4	53.8	3.46 (1.99-6.01)	52.0	26.9	0.57 (0.32-1.03)	9.0	9.9	0.87 (0.37-2.01)
2000	58.9	83.8	2.80 (1.62-4.85)	35.9	49.3	1.22 (0.75-2.01)	71.3	44.4	0.52 (0.32-0.87)	10.0	21.8	2.36 (1.20-4.64)
2002	75.8	80.5	0.95 (0.50-1.81)	34.0	53.2	2.27 (1.36-3.78)	45.7	31.2	0.33 (0.19-0.55)	10.6	25.8	3.16 (1.58-6.32)
2004	73.3	88.6	1.43 (0.71-2.89)	47.1	59.3	1.68 (1.01-2.86)	62.8	34.8	0.40 (0.23-0.70)	6.7	13.6	1.34 (0.62-2.93)
2006	77.8	85.3	1.29 (0.61-2.75)	45.4	63.2	1.91 (1.09-3.33)	56.5	29.4	0.51 (0.28-0.92)	7.4	17.4	1.31 (0.61-2.81)
2008	80.2	87.7	1.42 (0.67-3.03)	59.3	64.2	1.85 (1.05-3.24)	44.0	24.7	0.40 (0.22-0.72)	19.8	26.5	1.66 (0.87-3.17)
2010	88.8	85.4	0.81 (0.38-1.72)	46.4	65.3	2.30 (1.32-4.01)	60.0	32.7	0.42 (0.24-0.75)	11.9	23.3	2.56 (1.25-5.24)

*RII - relative index of inequality; CI - confidence interval; Adjusted for age and place of residence; x-data are not available;

Low education - Primary school or incomplete secondary school, High education - University.

**Table 4:** Educational inequalities in nutrition habits among women (prevalence (%) and RII*)

Years	Using vegetable oil for cooking			Using butter on bread			Drinking high-fat milk			Eating fresh vegetables daily		
	Education		RII	Education		RII	Education		RII	Education		RII
	Low	High	95% CI	Low	High	95% CI	Low	High	95% CI	Low	High	95% CI
1994	21.7	64.9	3.06 (1.78-5.26)	72.9	57.1	0.59 (0.35-0.99)	65.6	33.7	0.53 (0.30-0.93)	x	x	x
1996	48.9	82.9	3.34 (1.90-5.88)	50.0	59.8	2.20 (1.36-3.57)	55.4	27.9	0.52 (0.30-0.90)	7.4	6.8	1.21 (0.43-3.40)
1998	72.4	87.7	1.31 (0.68-2.51)	35.3	51.8	3.20 (1.97-5.19)	53.8	24.5	0.35 (0.20-0.59)	15.4	27.3	2.41 (1.33-4.38)
2000	76.6	92.7	1.99 (1.03-3.85)	31.2	56.9	5.07 (3.16-8.13)	72.5	43.3	0.56 (0.35-0.90)	14.9	22.4	1.14 (0.66-1.98)
2002	84.2	93.3	2.21 (1.01-5.17)	44.2	54.5	1.97 (1.24-3.13)	44.7	33.3	0.48 (0.30-0.77)	12.8	26.9	1.19 (1.15-3.50)
2004	80.7	91.8	1.75 (0.81-3.78)	45.6.	55.1	1.54 (1.02-2.48)	70.2	29.8	0.30 (0.18-0.51)	9.6	21.9	1.60 (0.86-3.00)
2006	81.7	94.2	2.91 (1.31-6.44)	44.5	63.6	2.01 (1.24-3.26)	68.2	22.3	0.20 (0.12-0.34)	18.3	31.8	1.65 (0.94-2.87)
2008	91.3	92.1	1.05 (0.42-2.61)	63.0	69.1	1.77 (1.09-2.88)	46.8	21.0	0.52 (0.31-0.90)	28.3	33.2	1.77 (1.06-2.95)
2010	91.4	96.9	1.54 (1.40-4.34)	38.8	67.5	3.25 (2.09-5.04)	54.3	25.8	0.45 (0.28-0.71)	13.8	29.6	2.75 (1.66-4.56)

*RII - relative index of inequality; CI - confidence interval; Adjusted for age and place of residence; x-data are not available;

Low education - Primary school or incomplete secondary school, High education - University.

Nutrition habits were related to the respondent's place of residence. The use of vegetable oil in cooking, daily consumption of fresh vegetables, and the usage of butter on bread were more common in cities than in rural areas (Table 5). The respondents in rural areas consumed high-fat milk more often than those living in cities.

**Table 5:** Age-adjusted prevalence (%) of given nutrition habits by place of residence (95% confidence intervals) (all study years combined)

Nutrition habits	Place of residence		
	City	Town	Village
**Men**	**n = 3118**	**n = 2011**	**n = 2226**
Using vegetable oil in cooking	77.0 (75.5-78.5)	74.2 (72.3-76.2)	61.1 (59.1-63.2)
Using butter on bread	55.7 (54.0-57.5)	47.3 (45.2-49.4)	49.4 (47.4-51.5)
Drinking high-fat milk	27.8 (26.2-29.3)	48.4 (46.2-50.6)	66.8 (65.8-68.7)
Eating fresh vegetables daily	16.1 (14.7-17.4)	14.0 (12.4-15.6)	12.2 (10.7-13.7)
**Women**	**n = 4500**	**n = 2813**	**n = 2474**
Using vegetable oil in cooking	88.0 (87.1-89.0)	85.3 (84.0-86.6)	70.5 (68.7-72.3)
Using butter on bread	56.8 (55.3-58.2)	50.6 (48.7-52.4)	48.0 (46.0-50.0)
Drinking high-fat milk	25.1 (23.8-26.3)	44.2 (42.4-46.1)	67.9 (66.0-69.7)
Eating fresh vegetables daily	23.0 (21.7-24.3)	19.0 (17.5-20.6)	16.3 (14.7-17.9)

The increase in the use of vegetable oil was more evident in villages, therefore differences by place of residence diminished in the most recent surveys (Tables 6, 7). The proportion of persons spreading butter on bread decreased in villages, but did not changed in cities. Since 2000, urban-rural differences became significant among women (Table 7). The same differences in men were the highest in 2000 and 2010. The differences in consumption of high-fat milk by living place diminished during the study period due to decreasing trends in consumption in rural areas. For both cities and villages, the frequency of daily consumption of fresh vegetables increased. Urban-rural differences in daily consumption of vegetables were small, but reached statistical significance in men in 2000 and 2006, and in women reached significance in 2004 and 2006 (Tables 6, 7).

**Table 6:** Differences in nutrition habits by place of residence among men (prevalence and adjusted OR*)

Years	Using vegetable oil in cooking			Using butter on bread			Drinking high-fat milk			Eating fresh vegetables daily		
	Place of residence		OR 95% CI	Place of residence		OR 95% CI	Place of residence		OR 95% CI	Place of residence		OR 95% CI
	Village	City		Village	City		Village	City		Village	City	
1994	19.6	42.6	2.85 (1.95-4.18)	73.1	68.1	0.97 (0.67-1.38)	69.8	21.8	0.12 (0.08-0.18)	x	x	x
1996	39.4	67.4	3.23 (2.31-4.51)	55.5	59.8	1.12 (0.81-1.54)	70.4	21.8	0.12 (0.08-0.17)	3.4	4.3	1.3 (0.58-2.92)
1998	60.0	75.7	2.01 (1.40-2.89)	31.6	41.8	1.29 (0.91-1.81)	65.3	17.6	0.12 (0.08-0.18)	12.3	10.0	0.78 (0.47-1.30)
2000	62.6	78.7	1.92 (1.36-2.71)	33.0	45.9	1.64 (1.12-2.25)	77.8	42.5	0.23 (0.17-0.33)	10.9	18.4	1.66 (1.06-2.60)
2002	76.8	82.4	1.31 (0.81-2.12)	48.6	49.8	0.97 (0.66-1.44)	63.5	33.5	0.28 (0.18-0.42)	17.5	18.5	1.06 (0.63-1.79)
2004	74.4	88.3	2.32 (1.43-3.74)	48.7	53.7	1.13 (0.79-1.61)	66.8	29.1	0.23 (0.16-0.33)	10.1	15.2	1.55 (0.90-2.67)
2006	79.2	87.4	1.96 (1.17-3.30)	50.6	58.5	1.18 (0.80-1.74)	64.3	26.8	0.22 (0.15-0.34)	8.9	18.6	2.28 (1.21-4.27)
2008	80.3	85.4	1.36 (0.84-2.22)	56.5	63.0	1.29 (0.89-1.88)	50.8	26.3	0.36 (0.24-0.52)	22.8	25.4	1.16 (0.75-1.78)
2010	83.1	88.0	1.62 (1.01-2.61)	34.6	65.4	1.47 (1.03-2.08)	62.2	26.4	0.23 (0.16-0.33)	17.6	18.8	0.98 (0.63-1.53)

OR - odds ratio; CI - confidence interval; OR city versus village; *Adjusted for age and education; x-data are not available

**Table 7:** Differences in nutrition habits by place of residence among women (prevalence and adjusted OR*)

Years	Using vegetable oil in cooking			Using butter on bread			Drinking high-fat milk			Eating fresh vegetables daily		
	Place of residence		OR 95% CI	Place of residence		OR 95% CI	Place of residence		OR 95% CI	Place of residence		OR 95% CI
	Village	City		Village	City		Village	City		Village	City	
1994	25.8	62.4	3.95 (2.87-5.46)	68.6	62.6	0.86 (0.63-1.17)	75.4	24.2	0.12 (0.09-0.17)	x	x	x
1996	43.8	83.4	5.58 (3.98-7.82)	47.8	53.7	1.18 (0.87-1.58)	72.1	22.2	0.12 (0.09-0.17)	4.4	6.6	1.52 (0.78-2.94)
1998	72.0	89.3	3.05 (2.02-4.60)	35.6	45.0	1.32 (0.95-1.81)	65.2	17.7	0.13 (0.09-0.18)	18.2	19.7	1.00 (0.67-1.48)
2000	77.0	91.6	2.81 (1.85-4.26)	27.0	46.0	1.97 (1.44-2.70)	83.7	37.5	0.13 (0.09-0.18)	17.9	20.8	1.15 (0.80-1.66)
2002	85.9	92.6	1.94 (1.06-3.56)	35.9	53.5	1.91 (1.28-2.85)	64.8	32.2	0.25 (0.17-0.38)	16.5	24.2	1.51 (0.91-2.50)
2004	82.9	93.2	2.72 (1.62-4.57)	47.1	57.1	1.44 (1.04-2.00)	79.4	26.3	0.10(0.07-0.15)	13.2	24.2	1.90 (1.23-2.95)
2006	85.4	92.0	1.61 (0.93-2.77)	51.2	62.8	1.42 (1.01-2.03)	68.8	24.5	0.18 (0.12-0.27)	13.7	30.7	2.62 (1.64-4.20)
2008	89.5	94.0	2.09 (1.14-3.82)	59.0	68.5	1.40 (1.00-1.98)	45.0	18.8	0.27 (0.19-0.39)	26.5	30.6	1.19 (0.82-1.73)
2010	91.2	93.3	1.26 (0.76-2.10)	52.3	63.8	1.35 (1.02-1.78)	53.4	20.1	0.24 (0.18-0.32)	20.5	27.8	1.29 (0.94-1.77)

OR - odds ratio; CI - confidence interval; OR city versus village; *Adjusted for age and education; x-data are not available

## Discussion

Our study has demonstrated some positive changes in the food habits of the Lithuanian adult population during the post-communist transition period. The use of vegetable oil in cooking and the frequency of consumption of fresh vegetables both increased. Conversely, the use of butter on bread decreased and the proportion of women drinking high-fat milk declined.

Several factors could explain these trends in food habits. Great changes occurred within the Lithuanian food market during this study period. Trade was liberalized; healthier food products, such as vegetable oils, soft margarines, low-fat dairy products, and a variety of fruits and vegetables became available. Affordable prices and good quality contributed to increases in consumption of these foods. Lithuanian food behaviours may have also been affected by health promotion campaigns and new health policies that emphasized the role of healthy nutrition in NCD prevention. For example, targets for healthy nutrition were included in the Lithuanian Health Programme (1998) [17]. In 2003, the Republic of Lithuania approved the State Food and Nutrition Strategy and Action Plan for 2003-2010 [18]. The main goal of this strategy was to reduce the prevalence of diet-related diseases and to improve the population's overall health. Among its priorities was increasing the accessibility and affordability of healthy food for all social groups. Food-based dietary guidelines were published to help the population make healthier choices [19]. These guidelines encouraged reduced fat consumption, especially from animal fat sources, limited consumption of meat products, and promotion of fruit and vegetable consumption.

Recently, the national diets of European countries became less distinct [20]. Mediterranean countries increased their consumption of meat, milk and sugar; meanwhile, some Northern countries decreased their intake of such foods and increased their fruit and vegetable consumption [21-26]. Dietary trends observed elsewhere in Central and Eastern European countries are similar to those found in Lithuania [10,11,27,28]. The increase in the availability of vegetable oils, margarines, fresh fruits and vegetables - even when out of season - was followed by rise in the consumption of healthier foods. Stimulated by the initiative of the WHO European Regional Office, many European countries developed their own policies on food and nutrition that contributed to favourable changes in nutrition habits of their populations [24,29,30].

Our study revealed that trends in nutrition habits varied between various socio-demographic groups. The increase in the use of vegetable oil was more evident among less educated people and those living in villages compared to the highly educated and urban residents. Increased availability of vegetable oils stimulated widespread replacement of lard-based cooking technique, which was traditionally used in food preparation, particularly among lower socio-economic groups. In 1987, 52.0% of the Lithuanian rural population used mostly lard in cooking [31]. During the study period, social differences in use of vegetable oil in cooking have diminished.

Our findings showed that trends in the type of fat spread on bread also differed by socio-demographic group. A greater proportion of less educated people and rural inhabitants replaced butter with margarine. Such trends might be explained by the increase in the price of butter over time. When margarines, which are considerably cheaper than butter, became available, the use of butter decreased among those with lower incomes. Data from previous studies illustrated that food prices influence food choice and that people of lower socio-economic position are the most susceptible to price fluctuation [32,33]. Moreover, cultural factors and the food-related beliefs of consumers are also important contributors to food choice [32,34]. For example, in Lithuania butter is considered an organic, healthy food. People are sceptical of replacing butter with margarine, which is seen as a processed, 'artificial' food, with negative impacts on health. The media supports such attitudes by providing misleading information about the health benefits of butter produced by Lithuanian farmers. The Lithuanian media may have a greater impact on highly educated people who use butter more often than the less educated. Similar educational patterns were observed in Russia, Latvia, Poland, and other countries with transition economies [10,11,35]. Meanwhile, the opposite association was found in Estonia and the Nordic countries [11,35]. Health education campaigns warning against butter as source of saturated fatty acids and recommending unsaturated fats might have influenced the behaviours of the highly educated in those countries.

According to our data, less educated and rural populations consumed high-fat milk most often. Social differences in milk consumption did not change over time. Such results are in line with other studies that reported an inverse association between consumption of high-fat milk and education level [36-38].

During the study period, daily consumption of fresh vegetables increased in all educational groups. However, this increase was most evident among the highly educated. In Lithuania, fresh vegetables and fruits are inexpensive in summer, but in spring and winter, when most fresh vegetables and fruits are imported, their prices rise. Our surveys were carried out in April and May, when availability of local vegetables was limited and prices were high. In order to increase consumption, the Lithuanian Government reduced VAT rate on fruits and vegetables in 2008; one year later, the VAT rate was restored because of the global economic crisis. According to comparison of surveys from nine European countries, a positive association between education level and vegetable consumption was related to the availability of vegetables [9]. This positive association was observed in countries with low availability and high prices for fruits and vegetables (Nordic and Baltic countries), whereas in countries where the availability and affordability were higher (France, Italy, Spain), the association was opposite. Northern countries do not have a tradition of consuming vegetables regularly. In those countries, consumption of fruits and vegetables is considered a 'modern' food habit. Higher socioeconomic groups are typically the first to choose these 'modern' foods [9].

In general, our study indicated beneficial dietary changes within the Lithuanian adult population. However, food habits of a large proportion of the Lithuanians do not meet healthy nutrition recommendations [19]. Therefore, Lithuanian Food and Nutrition Policy need renewal and supplementation to achieve the desired change in nutrition habits. The Ministry of Health, in collaboration with other government sectors, is developing the new Intersectorial Food and Nutrition Action Plan for 2012 - 2014 that aims to harmonise all activities dealing with nutritional problems of Lithuanians.

Several limitations of our study should be considered when interpreting our results. Our questionnaire included food-related questions that were used to identify the groups of people with different food habits. However, we were not able to measure amounts of consumed food or to calculate the nutrient content of respondent's diet. Validation studies of the questionnaire, using the 24-hour dietary recall as a reference method, revealed that data obtained by the two methods correlated well and simple food questions could be used to estimate 'high' and 'low' users of certain foods [39].

We used education and place of residence to characterize socio-economic groups. The level of education within a population increases over time; therefore, a comparison of educational differences in food habits over a period of 16 years may be biased. In Lithuania, the system of primary-secondary-higher education developed between the two world wars. In Soviet times, the education system was rather uniform. The development of Lithuania's current system started in the 1990s. Since 2003, it covers preschool, general, secondary, vocational, and higher (college and university) education. After reform of the vocational education system, college, as a higher education institution, was established. We grouped all respondents with college and vocational educations into one category, in order to maintain comparable educational categories throughout the study period. We were unable to study the effects of income on food habits; however, previous studies carried out in a Lithuanian population showed that educational level was directly associated with income [40]. The question about income appeared to be very sensitive to our respondents and was not included in the most surveys.

Furthermore, people belonging to higher social classes are more likely to be conscious of socially desirable eating habits and this might affect the reporting of nutrition habits [41]. Moreover, our findings may not be entirely representative, since non-response is often associated with unhealthy behaviours. The response rate tended to decrease in the most recent surveys. Therefore, it is possible that our results show a more favourable picture than if all selected samples were examined. We did not have data on non-respondents, but comparison of early and later respondents found only slight differences [42].

Despite the limitations described above, several major strengths of our study can be emphasized. First, it provides long-term trends in nutrition habits of Lithuanian adult population covering almost two decades of economic transition. Second, all cross-sectional surveys were carried out on nationally representative samples following the same methodology. Response rates were relatively high. Finally, to obtain maximal comparability, the questions regarding foods were asked in the same way in all surveys.

The Health Behaviour Monitoring has offered reliable data for the planning and evaluation of Lithuanian Food and Nutrition Policy. The future actions should be aimed at the increase of fresh vegetables consumption and reduction of high-fat dairy products intake targeting the most vulnerable groups of society.

## Conclusions

Our study showed an improvement in food habits of the Lithuanian adult population during the period 1994-2010. The consumption of animal fat declined, while the use of vegetable oil in cooking and consumption of fresh vegetables increased. In general, those with a higher level of education had healthier food habits than those with low education. The educational gradient in analyzed food habits, except the use of vegetable oil, enlarged. A higher proportion of the rural population compared to urban reduced their usage of butter on bread. However, drinking of high-fat milk was most prevalent in the rural population. These results highlight the need for future food and nutrition policies and health promotion programmes targeting the whole population, particularly those with lower education and living in rural areas.

## Abbreviations

NCD: noncommunicable diseases; CVD: cardiovascular diseases; SD: standard deviation; OR: odds ratio; CI: confidence interval; RII: relative index of inequality.

## Competing interests

The authors declare that they have no competing interests.

## Authors' contributions

JP and JK made substantial contributions to conception and design of the manuscript. ES and VK carried out statistical analysis. VK was involved in drafting of the manuscript. All authors read and approved the final version of manuscript.

## Pre-publication history

The pre-publication history for this paper can be accessed here:

http://www.biomedcentral.com/1471-2458/12/218/prepub
